# Combining mechanisms of action with behavior change techniques—theory-based development of an app promoting heating energy-saving behaviors

**DOI:** 10.3389/fpsyg.2025.1534014

**Published:** 2025-06-18

**Authors:** Mara Brandt, Sebastian Bamberg

**Affiliations:** ^1^Medical Assistance Systems Group, Medical School OWL, Bielefeld University, Bielefeld, Germany; ^2^Interactive Robotics in Medicine and Care, Medical School OWL, Bielefeld University, Bielefeld, Germany; ^3^Department of Social Sciences, University of Applied Sciences and Arts, Bielefeld, Germany

**Keywords:** stage model of self-regulated behavioral change, virtual agent, theory-based intervention development, mechanisms of action, behavior change techniques, theory and technique tool, menu-based, energy-saving behavior

## Abstract

**Introduction:**

There is an ongoing debate whether the currently used psychological interventions to motivate people to switch to more pro-environmental behavioral alternatives are effective. In the present paper the ‘theory and technique tool’ (TaTT) developed by the Human Behavior Change Project is used to demonstrate the theory-based development of a mobile app promoting heating energy saving behaviors.

**Methods:**

For this purpose, from the stage model of self-regulated behavioral change (SSBC) so-called Mechanisms of Action (MoA) are derived mediating the impact of the intervention on behavioral change. The TaTT is then used for linking these MoAs systematically with evidence based ‘behavior change techniques’ (BCTs).

**Results:**

In a next step, conceptual design ideas are developed as operationalizations of the included BCTs. In an experimental lab study, we test the effectiveness of one central conceptual design idea aiming to motivate participants to use intervention packages specially tailored to the needs which according to the SSBC an intervention has to target in that stage. The results, however, provide little empirical evidence that this design idea works as theoretically expected.

**Discussion:**

This finding underlines the importance of explicitly testing the ability of conceptual design ideas to activate theoretically proposed MoA-BCT links before the large-scale implementation of that intervention in a costly field study.

## 1 Introduction

Approximately two-thirds of global greenhouse emissions are directly or indirectly linked to household consumption (Ivanova et al., 2020). Changes in consumption patterns toward low-carbon alternatives, therefore, present a great potential for reducing greenhouse emissions. In the transport sector, for example, a car-free life or switching to a battery-electric vehicle with “green electricity” and avoiding air travel could reduce a person’s CO2 emissions by around 1.7 t per year (Ivanova et al., 2020). Unfortunately, there is still a debate about whether the currently available empirical evidence actually supports the effectiveness of behavioral change interventions used for this purpose (see, for example, the exchange between Nisa et al., 2019; van der Linden and Goldberg, 2020; Stern, 2020). In health psychology, a similar debate has led to the realization that more intellectual and empirical investment is urgently needed in designing effective, theory-based interventions. As a consequence, two research projects were established, focusing on the systematic theory-based intervention development: the Science Of Behavior Change (SOBC, Sumner et al., 2018) and the Human Behavior Change Project (HBCP, Michie et al., 2017). These research projects have provided important theoretical and methodological impulses on the subject of theory-based intervention development over the past decade.

**Figure 1:**
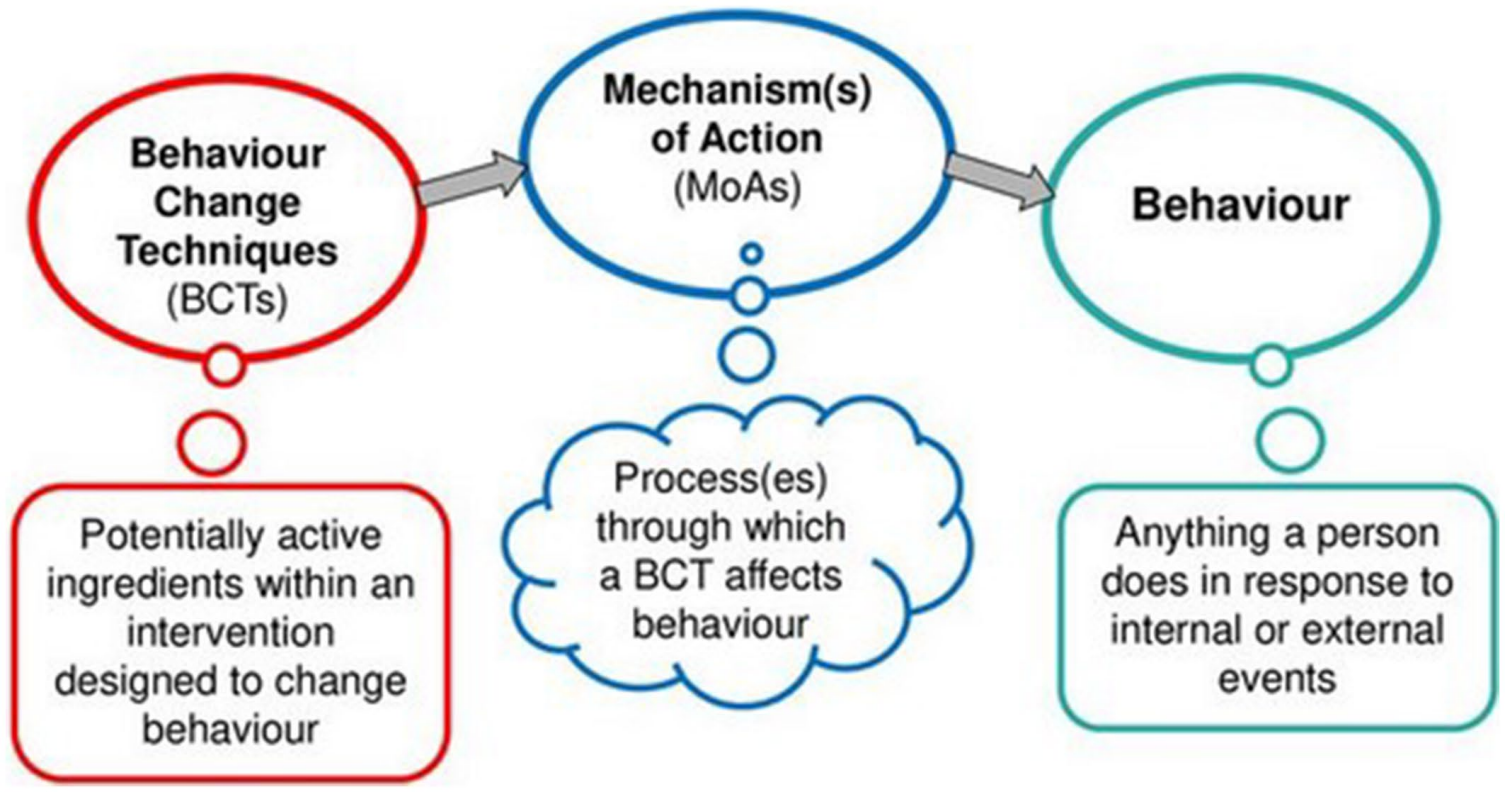
BCTs change behavior via mechanisms of action (adapted from Michie et al., 2016, p. 12).

Because there is currently no theoretical model that can understand and predict behavior change across all behavioral domains and contexts (Davis et al., 2014; Michie et al., 2014), both HBCP and SOBC take a more metatheoretical approach: theories of behavioral change are seen as ‘… a set of concepts and/or statements with specification of how phenomena relate to each other’, providing ‘… an organizing description of a system that accounts for what is known, and explains and predicts phenomena’ (Davis et al., 2014, p. 5). From this perspective, theories of behavior change are important for intervention development because they summarize why and how behavior change occurs, what is known about the role of specific constructs in this process, and propose both mechanisms of action and moderators of change along various causal pathways (Michie et al., 2018). Consequently, within the HBCP approach, behavior change theories play a central role in providing so-called ‘mechanisms of action’ (MoAs), which are defined as processes that mediate the effect of interventions on behavior change. On the other hand, in the HBCP approach, the active components of an intervention are referred to as “Behavior Change Techniques” (BCTs). BCTs are defined as an “observable, replicable, and irreducible component of an intervention designed to alter or redirect causal processes that regulate behavior” (Michie et al., 2013). The BCTs cause changes in the MoAs, which in turn cause behavioral changes (Figure 1).

Thus, according to the HBCP approach, the core of systematic intervention development consists of the theory-based development of so-called BCT-MoA links. An improved understanding of the links between BCTs and MoAs can facilitate the development of more effective interventions and improve the ability to explain how effective interventions bring about change. Most behavior change interventions use combinations of multiple BCTs, that is, the active ingredients within an intervention that lead to behavior change. The use of multiple BCTs within an intervention does not in itself necessarily increase intervention effectiveness; however, interventions that use a combination of BCTs aligned with a behavior change theory have been associated with increased intervention effectiveness (Dombrowski et al., 2012). Therefore, thinking systematically about how different BCTs work synergistically could improve our ability to examine associations between theory and intervention effectiveness.

Within the context of this discussion, the development of the BCT Taxonomy v1 (BCTTv1; Michie et al., 2013) was an important step. The BCTTv1 is a classification system for systematically specifying intervention components in terms of 93 BCTs organized into 16 groupings (Michie et al., 2013). In a methodologically similar process, Michie and colleagues (e.g., Michie et al., 2018; Carey et al., 2019) extracted a taxonomy of 26 MoAs. Of the 26 MoAs, 14 stemmed from the theoretical domains framework (Cane et al., 2012) and the remaining 12 MoAs included the most frequently occurring MoAs from 83 behavioral theories documented in the research literature (Michie et al., 2018; Carey et al., 2019). In two studies, both taxonomies were systematically linked: Connell et al. (2019) conducted an expert consensus study using the Delphi approach to link the most frequently occurring BCTs with the 26 MoAs (Connell et al., 2019); Johnston et al. (2020) conducted a triangulation study drawing on the results of the first studies to produce the so-called heat map classifying the BCT-MoA links into those with evidence for a “link,” “inconclusive,” “non-link,” and “no evidence.” This classification is based on an analysis of the results of 277 evaluation studies. By creating the web-based theory and technique tool (TaTT^1^), Michie and colleagues (Michie et al., 2018; Carey et al., 2019; Connell et al., 2019) have made the results of the above-described research process easily accessible to practitioners. Thus, intervention designers and evaluators can use the TaTT as a starting point for their intervention development process.

However, while MoA-BCT relationships can be systematically derived using TaTT, the concrete operationalization of a specific BCT in the context of an intervention project remains a primarily creative task. More precisely, this task consists of translating the relatively abstractly formulated BCTs into practical intervention elements used to influence the MoAs in such a way that the persons targeted by the intervention change their behavior or preserve this behavioral change.

## 2 The present study

Inspired in particular by the HBCP approach, the present study aims to use the theory-based intervention development approach in the area of promoting environmentally friendly behavior. We view applying the HBCP approach as a promising strategy to move forward in the abovementioned debate about how to effectively identify interventions that promote pro-environmental behaviors. More precisely, we apply the HBCP approach within the context of developing a tablet-based app that promotes heating energy-saving behaviors (e.g., lowering the temperature at night or if away for a long time, shock ventilation, and installing electronically controlled thermostats). Because 70% of a household’s fossil energy use relates to heating, interventions aiming to motivate consumers to use heating energy carefully have a high potential for reducing a household’s greenhouse emissions. The aim of the app is to motivate people to find out about their way to save heating energy, form an intention to actually implement selected heating energy-saving behaviors, implement this intention, and give them the opportunity to get feedback on the effects of these behavioral changes on their actual heating energy consumption.

In the first theoretical section of the article, we present the stage model of self-regulated behavioral change (SSBC) proposed by Bamberg (2013). We use the SSBC for identifying central mechanisms of action (MoAs) assumed to be associated with changes in pro-environmental behaviors. In the second conceptual section, we apply the web-based theory and technique tool (TaTT; Carey et al., 2019) to identify and select evidence-supported BCT-MoA links. In the third section, we develop design ideas for operationalizing the BCTs in the app. In the fourth empirical section, we present a lab-based approach for testing *a priori* the validity of assumptions derived from the conceptual framework. However, instead of testing the whole behavior-MoA-BCT links presented in Figure 1, the study focuses on a central assumption underlying the SSBC: As a stage model, it assumes that people in different stages of the behavioral change process need and prefer different intervention packages. The aim of the study is, therefore, to empirically test whether preferences for intervention packages differ between participants at different stages in the theoretically expected manner.

## 3 The stage model of self-regulated behavioral change

**Figure 2:**
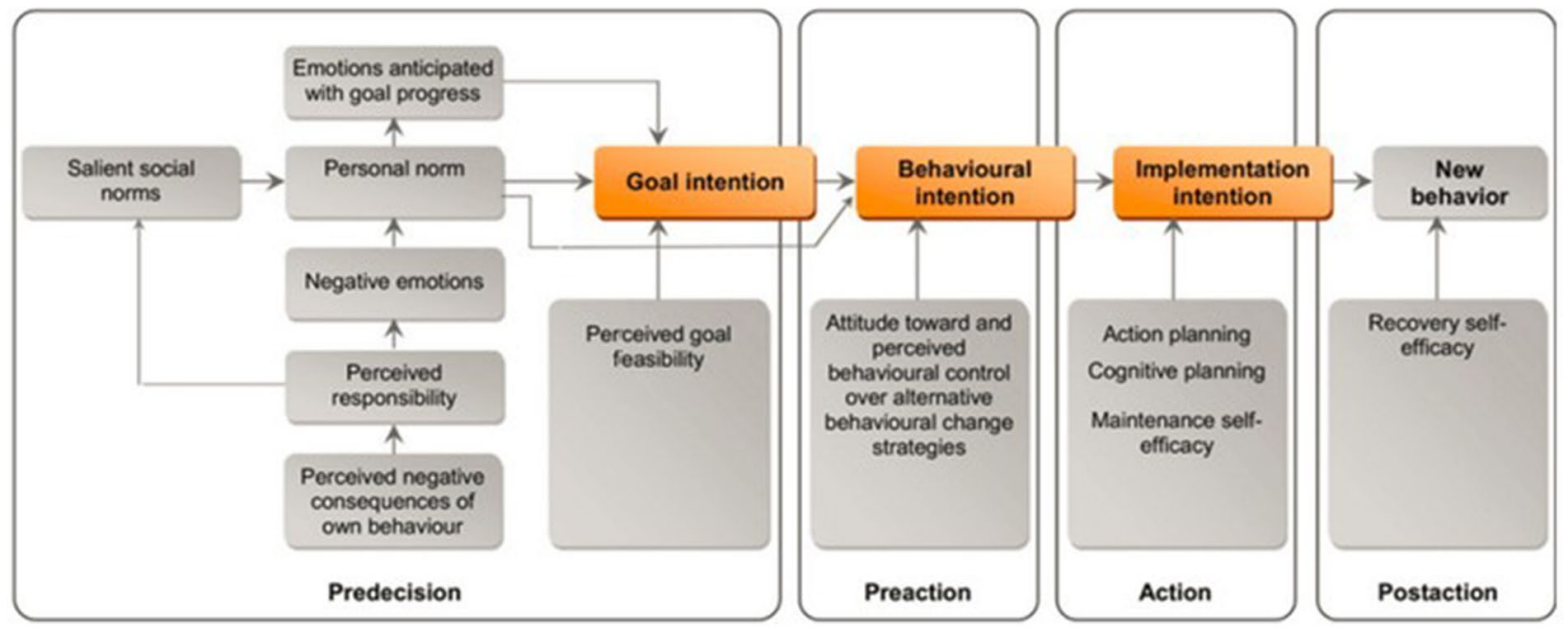
The stage model of self-regulated behavioral change (SSBC, Bamberg, 2013, p. 153).

In the last decades, stage models such as the Transtheoretical Model of Behavioral Change (TTM, Prochaska and Velicer, 1997) have become increasingly popular; however, they are also heavily criticized theoretical approaches to explain behavioral change. In addition to the question of whether behavioral change actually occurs in stages, the number as well as the theoretical demarcation and operationalization of these stages is controversial (e.g., West, 2005). Responding to this criticism, Bamberg (2013) developed the stage model of self-regulated behavioral change (SSBC). The SSBC combines the model of action phases (MAP; Gollwitzer, 2012) with other more static theories, such as the theory of planned behavior (Ajzen, 1991) and the norm activation model (Schwartz and Howard, 1981). Thereby, it aims to extend previous stage models by considering both the dynamic, longitudinal processes that reflect multiple stages of decision-making, as well as the factors influencing each of those single decisions or stage transitions. Figure 2 presents the SSBC graphically.

In the first stage, the predecision stage, the current behavior needs to be perceived as problematic, leading to an intention to reduce this behavior and, subsequently, the negative consequences resulting from it (goal intention). Variables assumed to influence the formation of such a goal intention are primarily taken from the norm activation model (NAM; Schwartz and Howard, 1981). In the next stage, the preaction stage, an individual has to choose an alternative behavior to achieve the goal of reducing their current problematic behavior formed in the predecision stage. The formation of such a behavioral intention could be modeled via the theory of planned behavior (TPB; Ajzen, 1991). Next, in the actional stage, the new behavior has to be implemented in everyday life, determined by the strength of a person’s implementation intention (Gollwitzer, 1999), which depends on a person’s planning abilities and maintenance self-efficacy. Finally, in the postaction stage, the strength and weaknesses associated with the performance of the new behavior are validated. Preventing relapse in critical situations is also an important task in the postaction stage.

A systematic review of studies applying the SSBC, published by Keller et al. (2019), includes 10 studies on environmental topics, such as travel mode choice and smart meter use, published between 2013 and 2018, six of which employed a cross-sectional design, three an interventional design, and one a correlational longitudinal design. The cross-sectional and longitudinal studies generally support the model, although there are some irregularities that warrant further investigation.

The SSBC was explicitly developed as a conceptual framework for guiding systematic intervention development. There is one practical important implication of considering behavioral change as a transition process through different stages: There is no single intervention for all. Instead, specific intervention packages have to be developed to meet the needs and barriers of persons in each specific stage. For instance, interventions targeting persons in the predecision stage are more likely to be successful if they concentrate on providing information that can increase both problem awareness and perceived personal responsibility. Moreover, interventions activating social and personal norms are likely to be important in this stage. Individuals who already have set a goal-intention (preaction stage) need information concerning the availability and the pros and cons of different behavioral alternatives. Individuals who intend on switching to an alternative behavior (actional stage) probably benefit most from interventions supporting the implementation and initiation of this intention, e.g., detailed behavioral planning. Individuals who have already implemented a new behavior (postaction stage) benefit from interventions that provide feedback on the behavioral consequences achieved in maintaining this new behavior. At the same time, these interventions help reduce relapses.

## 4 Using the web-based TaTT for systematically linking SSBC MoAs and BCTs

For applying the TaTT, in the first step, we have to translate the construct labels used by the SSBC into the more general MoA labels used by the TaTT. In the second step, for the SSBC constructs thus translated into the TaTT “language,” we used the TaTT app to select MoA-BCT links. This selection process was steered by two criteria: the links should be supported by empirical evidence, and the BCTs should be potentially applicable within the context of developing an app for promoting heating energy-saving behaviors.

### 4.1 Method

The selection process was conducted by the two authors. In the first step, each author separately decided how to translate the SSBC constructs into the MoA labels used by the TaTT. Then, disagreements were discussed and solved. In the second step, each author separately searched the TaTT for evidence-based links between the MoAs included in the SSBC and specific BCTs. In three iterative rounds, the authors compared their results, discussed disagreements, tried to solve them, and then repeated their rating.

### 4.2 Results

Columns 1 and 2 of Table 1 report the SSBC stages and the names of the stage-specific SSBC constructs selected as targets of the planned app. Column 3 presents the results of the first step, which involves translating the SSBC construct into the names of the respective TaTT-MoAs. Column 4 presents the results of the evidence-based MoA-BCT links resulting from information reported in the TaTT. As can be seen in Table 1, column 4, from this procedure for persons in the predecision stage, a package includes the following four BCTs ‘5.3. Information about social and environmental consequences’, ‘1.6. Discrepancy between current behavior and goal’, ‘6.2. Social comparison’, and ‘1.3. Goal setting (outcome)’ results. This BCT package is linked with the three MoAs ‘social norms’, ‘perceived negative consequences of own behavior’, and ‘goal intention to reduce heating energy consumption’, postulated by the SBCC as important MoAs for promoting in the predecisional stage doubts about the adequacy of current heating behaviors.

**Table 1:** Using the TaTT for linking MoAs derived from the SSBC with specific BCTs.

SSBC stage	SSBC constructs	TaTT MoAs	TaTT BCTs	Design operationalization	Design idea for app
Predecision	Perceived negative consequences of own behavior	Knowledge, beliefs about consequences, attitude toward the currently used behavioral option	5.3.^1^ Information about social and environmental consequences	Show the CO2 emissions of the own energy consumption	Menu “Current consumption”: Line graphs show the own heating energy consumption in equivalent CO2 emissions, View “General goal setting”: Possible energy savings of the desired energy saving goal are displayed in CO2 emissions, Euros, and kWh
	Goal intention	Goal setting, behavioral regulation	1.6. Discrepancy between current behavior and goal	Show the current heating energy consumption and the set goal value; show the current heating energy consumption in comparison to an optimal value	Line graphs show the heating energy consumption in the own apartment in comparison to a line showing an optimally achievable value. A progress bar displays the heating energy consumption in relation to the set general energy-saving goal
	Social norm	Social norms	6.2. Social comparison	Show the own energy consumption in comparison with similar neighbors	View with bar charts shows the own heating energy consumption in comparison with similar neighbors
	Goal intention	Goals	1.3. Goal setting (outcome)	Ask the user to set a general heating energy saving goal in terms of saved kWh in the next month or next heating period	Floka prompts the user to set a general energy saving goal, view provides the possibility to set a general energy-saving goal in kWh for a defined time period
Preaction	Perceived negative consequences of own behavior	Knowledge, beliefs about consequences, attitude toward the behavior	5.3. Information about social and environmental consequences	Provide behavior-specific information about the environmental consequences of saving energy in this way	Floka informs the user about the environmental consequences of most of the energy-saving tips in the details for each tip.
	Perceived goal feasibility, perceived behavioral control	Knowledge, skills, beliefs about capabilities	4.1. Instruction on how to perform behavior / 6.1. Demonstration of the behavior	The user is informed about the needed steps to perform a specific behavior (demonstration with images/videos)	Information about each energy-saving tip is provided by Floka in speech and/or image form. For most of the tips, additional implementation details can be viewed.
	Attitude toward new behavioral options	Attitude toward the behavior	9.1. Credible source	Show energy-saving tips from a credible source, use a trusted agent to provide the information	The used virtual agent Floka is known to be developed by our research team, and Co2online.de is used as a credible source for energy-saving tips.
	Behavioral intention	Intention, goals Intention	1.1. Goal setting (behavior) 1.9 commitment	Provide the possibility to set specific behavior as a goal with very specific terms and explicit commitment	Each tip presentation ends with a dialog to set an appropriate behavioral goal. Links to view provide the possibility to set behavior as a goal for 1 week or 1 month. The user has to agree to very specific terms for the goal setting “I will do xy for the next week/month” and commit to it with a button press.
Action	Perceived behavioral control, implementation intention	Beliefs about capabilities, behavioral cueing, beliefs about capabilities	1.2. Problem-solving/1.4 Action planning/9.3 Comparative imagining of future outcomes	Help the user to anticipate problems in achieving their goals and provide help on how to overcome them by planning detailed steps. Give the user the opportunity to imagine the performance of the new/wanted behavior and imagine the best outcomes.	The WOOP dialog and view provide input text fields to anticipate problems for specific goal behaviors and plan strategies to overcome them; also, imagination of the wanted behavior and best outcomes is prompted.
	Perceived behavioral control	Knowledge, skills, beliefs about capabilities	4.1. Instruction on how to perform behavior/6.1. Demonstration of the behavior	Show concrete steps for the user to achieve the wanted behavior, demonstrated with images/videos.	Implementation details for most of the energy-saving tips are provided as speech and/or image
	Behavioral intention	Motivation, Feedback Processes, goals	2.2. Feedback on behavior/1.6. Discrepancy between current behavior and goal/7.1. Prompts/cues	Provide feedback about the current status of the user’s set goals and show deviations of the behavior from their goals. Support with reminders and timely notifications if unwanted behavior is detected.	Menu “My goals” shows the progress of each set behavior goal with a color-coded status display. Messages/notifications to remind the user of their set behavioral goals with a status update Notifications if sensor data shows that the values (i.e., temperature) are deviating from the desired values
	Social norm	Social influence	3.2 Social support (practical)	Support the user with reminders of their goals and timely notifications if unwanted behavior is detected	Messages/notifications to remind the user of their set behavioral goals with a status update, notifications if sensor data shows that the values (i.e., temperature) are deviating from the desired values
Postaction		Goals, Motivation, feedback processes,	1.6. Discrepancy between current behavior and goal/2.2 Feedback on behavior/2.7 Feedback on outcome(s) of behavior	Tell/show the user, if their current behavior is not in line with their set goal(s); show the user if their goal is not achieved	Menu “My goals” shows the current status of the set goals Notifications inform about the current status of the set goals. Floka and goal closing view shows the achievement status of the set goal
		Feedback processes	6.2. Social comparison	Show how the users’ efforts to save energy pay off in comparison with similar neighbors	The own energy consumption is compared to the neighbors with a 5-star rating and additionally shown as bar charts
	Attitude toward behavior	Beliefs about consequences	10.10. Reward (outcome)	Award the user with a trophy for a successful behavior change	Goal completion view shows a trophy if the user achieved the goal; all trophies are shown on the “Trophy collection” view
		Goals	1.5. Review behavior goal(s)	Check the achievement of the set behavioral goal(s) and mark them as completed	Floka informs the user about the completion status of the behavior goal, and users can mark them as successfully/not successfully completed
		Goals	1.7. Review outcome goal(s)	Check the achievement of the set general energy-saving goal	Progress bar on the “My goals” view shows the achievement status of the set general energy-saving goal
		Beliefs about capabilities	15.3. Focus on past success	Encourage them to keep up the good work with another energy-saving goal; show the already achieved energy-saving goals in a trophy collection	Floka encourages the user to keep up the good work by setting another energy-saving goal. View “Trophy collection” shows collected trophies for already achieved goals

^1^The numbers (e.g., 1.5) in column 2 refer to the numbers used by BCT Taxonomy v1 to identify a specific BCT.

For persons in the preaction stage, a package including the six BCTs ‘5.3. Information about social and environmental consequences’, ‘4.1. Instruction on how to perform behavior’, ‘6.1. Demonstration of the behavior’, ‘9.1. Credible source’, ‘1.1. Goal setting (behavior)’, and ‘1.9 Commitment’ is rated as linked with the three MoAs ‘attitude toward energy saving behavioral options’, ‘perceived behavioral control over energy saving behavioral options’, and ‘behavioral intention to perform energy saving behavioral options’, postulated by the SBCC as important MoAs in the preaction stage for promoting participants’ intention to change their current heating behaviors.

For persons in the action stage, a package including the nine BCTs ‘1.2. Problem solving’, ‘1.4 Action planning’, ‘9.3 Comparative imagining of future outcomes’, ‘4.1. Instruction on how to perform behavior’, ‘6.1. Demonstration of the behavior’, ‘2.2. Feedback on behavior’, ‘1.6. Discrepancy between current behavior and goal ‘, ‘7.1. Prompts/cues’, ‘3.2 Social support (practical)’ is rated as linked with the three MoAs ‘action planning’, ‘cognitive planning’, and ‘maintenance self-efficacy’ postulated by the SBCC as important MoAs for implementing the intended behavioral change process.

For persons in the postaction stage, a package including the eight BCTs ‘1.6. Discrepancy between current behavior and goal’, ‘2.2 Feedback on behavior’, ‘2.7 Feedback on outcome(s) of behavior’, ‘6.2. Social comparison’, ‘10.10. Reward (outcome)’; ‘1.5. Review behavior goal(s)’, ‘1.7. Review outcome goal(s)’, and ‘15.3. Focus on past success’ is rated as linked with the three MoAs ‘maintenance self-efficacy’, ‘recovery self-efficacy’, and ‘evaluation of outcomes associated with adopted new behaviors’ postulated by the SBCC as important MoAs for evaluating and maintaining the behavioral change process.

## 5 Developing design ideas for operationalizing BCTs implemented in the app

As stated above, operationalizing a specific BCT in the context of a concrete intervention project is primarily a creative task, however, based on knowledge of the scientific intervention literature. In the present case, this task consists of translating the generally defined BCTs into concrete ideas of how to operationalize them in the context of an app-based intervention. This should be done in such a way that they are actually able to change the constructs assumed to underlie participants’ current heating behavior and their intentions to change this behavior and/or maintain this change.

### 5.1 Method

In the present project, we started the creative process of developing BCT operationalizations by searching the empirical intervention literature for examples of already existing BCT operationalizations in the field of pro-environmental behaviors (e.g., Abrahamse, 2019, 2020). Before this knowledge background, we conducted a creative ideas producing brainstorming workshop with the whole research group. Three members of the research group are experienced intervention developers.

### 5.2 Results

In Table 2, columns 5 and 6 document the results of this brainstorming process. For example, we operationalize the BCT ‘5.3.1 Information about social and environmental consequences’ taken from the TaTT in the context of heating energy saving as ‘Show the CO2 emissions of the own energy consumption’. The creative solution to implement this BCT in an app is ‘Line graphs show the own heating energy consumption in equivalent CO2 emissions’.

**Table 2:** Assignment of main menu buttons to SSBC stage and BCTs.

SSBC stage	BCT	App ‘Buttons’
Predecision	5.3. Information about social and environmental consequences/1.6. Discrepancy between current behavior and goal	“My energy consumption”
	6.2 Social comparison	“Comparison with neighbors”
	1.3 Goal setting (outcome)	“Set energy saving goal”
Preaction	5.3 Information about social and environmental consequences/4.1 Instructions on how to perform the behavior/6.1 Demonstration of the behavior/9.1 Credible source / 1.1 Goal setting (behavior)	“Energy saving tips”
Action	1.2. Problem-solving/1.4 Action planning/9.3 Comparative imagining of future outcomes/15.2 Mental rehearsal of successful performance	“Plan energy saving behavior”
	2.2. Feedback on behavior/1.6. Discrepancy between current behavior and goal/7.1. Prompts/cues	“My goal progress”
Postaction	1.6. Discrepancy between current behavior and goal/2.2 Feedback on behavior/2.7 Feedback on outcome(s) of behavior/1.7. Review outcome goal(s)	“My goal progress”
	6.2. Social comparison	“Comparison with neighbors”

## 6 Testing stage-specific preferences for different intervention packages

In the following sections, we present a laboratory-based experimental approach that allows us to test the empirical validity of theoretically derived intervention design ideas during the intervention development phase. As stated in Section 2, in the case of this study, we conducted a lab experiment with the aim to test the central SSBC idea that a person’s stage membership determines their preference for specific intervention elements. Specifically, this means that we need a design idea of how we can make it clear to the user on the app’s home screen which stage-specific intervention packages are included in the app. Against the background of the SSBC, Sunio and Schmöcker (2019) assume that users then select the app element that they believe provides the information relevant to their current situation (stage membership). The central task of app intervention developers is, therefore, to develop a design idea that signals to potential users in a simple and clear way what kind of information/services they can expect from selecting a particular app element. If empirically validated, the advantage of this approach would be that no complex preliminary classification is required when conducting a stage-based intervention study. Instead, users are provided with stage-specific interventions from which they can select the one that best suits their needs.

### 6.1 The design idea of how to signal the stage-specific significance of intervention elements

**Figure 3:**
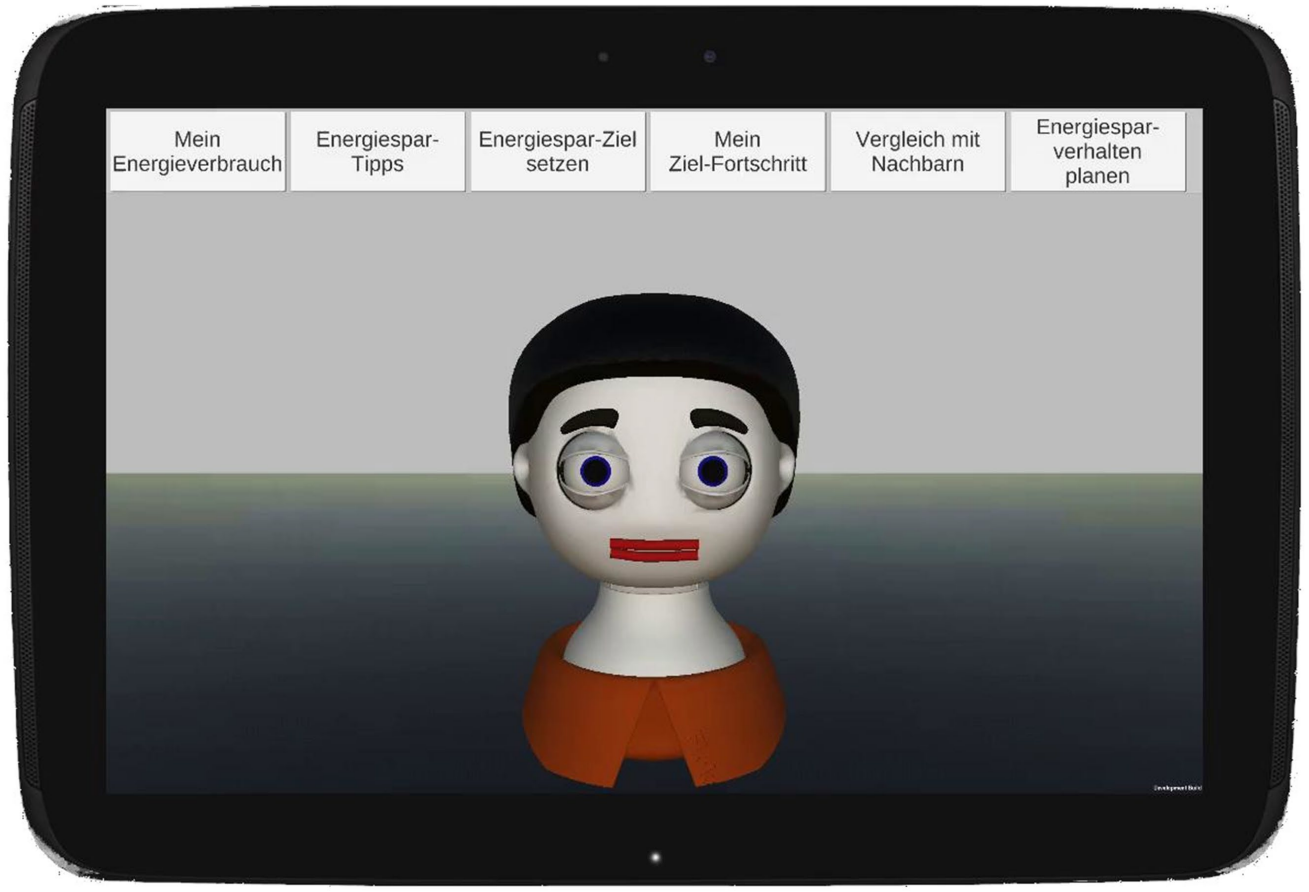
App home screen with the virtual agent Floka and the main menu.

Figure 3 shows the conceptual design idea developed to test this theoretical idea. The six buttons “My Energy Consumption,” “Energy Saving Tips,” “Set Energy Saving Goal,” “My Goal Progress,” “Compare with Neighbors,” and “Plan Energy Saving Behavior” are intended to provide users with the information that they can use to decide whether the intervention elements summarized under one button meet their stage-specific needs.

Furthermore, a virtual agent named Floka is used to introduce the user to the functions summarized under a button. Table 3 reports which BCTs the virtual agent mentions, when presenting and explaining the six app buttons (in the appendix the dialogs are documented, the German speaking video could be seen under https://youtu.be/Oe2VH8d2RMY).

**Table 3:** Items used for stage classification.

Item	Stage classification
At the moment, saving heating energy is not an issue for me. I see no reason to change anything.	Predecisional
I would like to reduce my heating energy consumption, but at the moment, I do not see any way for me to do so.	Predecisional
My goal is to use less heating energy. But I do not know what I can do specifically in my household	Preactional
I already know exactly how I can save heating energy in my household. However, I have not yet managed to put this intention into practice.	Actional
Saving heating energy is important to me, so I have already done a lot to save heating energy in my household and intend to implement other energy-saving measures.	Postactional

### 6.2 Hypotheses

From the SSBC, we derive the hypotheses on how stage membership determines persons’ preference for the six app buttons:

- H1. Persons assigned to the predecision stage prefer most frequently the buttons ‘My energy consumption’ (H1a), ‘Comparison with neighbors’ (H1b), and ‘Set energy saving goal’ (H1c).
- H2. Persons assigned to the preaction stage prefer most frequently the button ‘Energy saving tips’.
- H3. Persons assigned to the action stage prefer most frequently the buttons ‘Plan energy saving behavior’ (H3a) and ‘My goal progress’ (H3b).
- H4. Persons assigned to the postaction stage prefer most frequently the buttons ‘My goal progress’ (H4a) and ‘Comparison with neighbors’ (H4b).

### 6.3 Method

#### 6.3.1 Procedure and measures

Data collection followed APA guidelines for the ethical conduct of research and included informed consent. After reading the introduction explaining the study’s aim (evaluating the relevance and attractiveness of a planned intervention aiming to motivate participants to save energy and related information), participants first reported their age and gender. Then, they completed a measure used for classifying their current stage membership. In the next step, they viewed the above-presented video explaining the six app buttons with the help of the virtual agent Floka. In the final step, participants filled out three measurement instruments that we used to record participants’ preferences for the six app buttons shown in the video. These three preference measures consist of one forced choice task and two Likert scales asking participants to rank the perceived personal relevance as well as the attractiveness of the six app buttons presented in the video.

The measure for classifying participants’ stage membership was developed originally by Bamberg (2013) in the context of reducing car use. For the present study, this measure was adapted to the domain of heating energy saving. The measure consists of a forced-choice task asking participants to choose from the five statements presented in Table 3, the one that best describes their current heating behavior and their plans for the future. For example, participants who select item 1 or 2 are assigned to the predecisional stage.

After completing the stage classification task, participants viewed the video and completed one control item, assuring that participants have actually observed the video (‘In the video you just watched: What is the name of the character who spoke in the energy saving app?’). Participants were only included in the study if they answered this attention control question correctly. As a final step, all participants answered the three preference measures. The first measure consists of a forced choice task: ‘Which of the six buttons presented in the video would you choose if you were using this app in your current situation? Please select the feature that you find most helpful in your current situation.’ Participants then could select only one of the presented six app buttons ‘My current energy consumption’, ‘Energy saving tips’, ‘Set an energy saving goal’, ‘My goal progress’, ‘Comparison with neighbors’, and ‘Plan energy saving behavior’. The other two measures asked participants to judge the perceived personal relevance (Not relevant at all = 1; very relevant = 5) and perceived personal attractiveness (Not attractive at all = 1; very attractive = 5) of the same six app buttons.

#### 6.3.2 Experimental context

The online experiment for testing the hypotheses was conducted in December 2021. In December 2021, not only did rents continue to rise in Germany but also the prices for natural gas, which is used to heat 70% of all German households. Therefore, tenants should be particularly interested in saving heating costs. December 2021, on the other hand, was significantly too warm in Germany (up to 17 degrees at the end of the month), which may have reduced interest in energy-saving tips.

#### 6.3.3 Participants

The participants in the experiment came from the Germany-wide access panel of a commercial online market research institute. Because the experiment was planned as a pre-study of a main study with tenants as the target group, only tenants were surveyed in the online experiment. The specific place of residence was not recorded. To be accepted as a study participant, a person has to be older than 17 years and has to live in a rented flat. All included participants fulfill these two eligibility criteria. Participation in the survey was compensated with €2.50. During the sampling process, the measure presented in Table 3 was used for classifying participants into the four SSBC stages. The goal was to acquire an equal number of participants for each of the four stages, large enough (n = 120) to have enough statistical power to conduct pairwise tests. For the SBCC stages ‘predecision’ and ‘postaction’, this goal was reached quickly. Consequently, we stopped acquiring participants assigned to these two stages and included only participants classified as being in the preaction and action stages. Using this procedure results in a total sample of 507 participants: 131 (25.9%) of the 507 participants were assigned to the pre-decisional stage, 129 (25.4%) to the pre-actional stage, 121 (23.4%) to the actional stage, and 126 (24.9%) to the post-actional stage. Of the 507 participants, 264 define themselves as female and two (0.4%) as neither female nor male. The average age is 49 years (median, range 18–86 years, SD = 14.9 years).

### 6.4 Results

#### 6.4.1 Results of the forced choice preference task

Separately for the four SSBC stage groups, Table 4 presents participants’ decisions in the forced choice task (‘Please select the feature that you find most helpful in your current situation.’).

**Table 4:** Stage-dependent preference for the app buttons (force choice task, absolute number in brackets).

App button	Predecisional	Preactional	Actional	Postactional
My current energy consumption (Predecision)	46% (60)	28% (36)	36% (43)	42% (53)
Energy saving tips (Preaction)	28% (36)	49% (63)	33% (40)	31% (39)
Set an energy-saving goal (Predecision)	10% (13)	10% (13)	10% (12)	10% (12)
My goal progress (Action, Postaction)	4% (5)	5% (6)	8% (10)	5% (6)
Comparison with neighbors (Predecision, Postaction)	3% (4)	2% (3)	3% (4)	4% (5)
Plan energy-saving behavior (Action)	10% (13)	6% (8)	10% (12)	9% (11)

Differences in frequencies reported in the 6×4 contingency table were analyzed using the ꭓ2 test. The test results indicate only the choice of two app buttons ‘My current energy consumption’ [χ2(3) = 10.17, *p* < 0.05] and ‘Energy saving tips’ [χ2(3) = 15,20, *p* < 0.05] significant differences between the four stage groups: Compared to the other three stage groups, participants diagnosed as belonging to the predecisional stage choose most frequently the app button ‘My current energy consumption’. Compared to the other three stage groups, participants diagnosed as belonging to the preactional stage choose most frequently the app button ‘Energy saving tips’. These results provide some empirical support for H1a and H2. All other six (sub)hypotheses concerning stage-specific button preferences, however, are not empirically supported by the forced choice task.

In the next step, we analyzed how participants perceived the personal relevance and attractiveness of the six app buttons. Explanatory factor analysis indicates that both measures used for assessing the relevance as well as attractiveness are unidimensional (promax method, 55.4% common variance of relevance scale, 60.6% common variance of attractiveness scale) and consistent (Cronbach’s *α* of relevance measure = 0.82; of attractiveness measure = 0.85). Furthermore, participants’ ratings of the relevance and attractiveness of the app buttons correlate quite high: For the button ‘Set an energy saving goal’, the correlation of both aspects is the lowest (*r* = 0.70) and for the aspect ‘Comparison with neighbors’, it is the highest (*r* = 0.82). However, additional factor analysis does not confirm that both scales load on a common dimension. For this reason, we analyze both measures separately.

Table 5 presents separately for the four stage groups participants perceived personal relevance of the six app buttons.

**Table 5:** Perceived relevance for the app buttons for the four stage groups separately.

App button	Predecision		Preaction		Action		Postaction	
	*M*	SD	*M*	SD	*M*	SD	*M*	SD
My current energy consumption	3.80	1.18	4.36	0.83	4.32	0.91	4.53	0.82
Energy saving tips	3.62	1.14	4.29	0.87	3.98	1.05	4.06	1.12
Set an energy-saving goal	3.34	1.09	3.74	0.96	3.72	1.04	3.73	1.16
My goal progress	3.32	1.12	3.64	0.92	3.77	1.01	3.81	1.14
Comparison with neighbors	2.43	1.36	2.30	1.23	2.69	1.32	2.48	1.46
Plan energy-saving behavior	3.20	1.20	3.62	1.07	3.49	1.07	3.62	1.20

A multivariate analysis of variance (MANOVA) was used to compare the perceived relevance of the six app buttons across the four SSBC stages. The multivariate result was significant for the SSBC stage, Pillai’s Trace = 0.131, *F* = 3.79, df = (18,1500), *p* < 0.01, indicating significantly different means of the perceived relevance of the six buttons across the four SSBC stages. The univariate F tests showed that there was a significant difference between the stages for the buttons ‘My current energy consumption’, *F* = 14.93, df = (3,503), *p* < 0.01, ‘Energy saving tips’, *F* = 9.11, df = (3,503), *p* < 0.01, ‘Set an energy saving goal’, *F* = 4.81, df = (3,503), *p* < 0.01, ‘My goal progress’, *F* = 5.74, df = (3,503), *p* < 0.01, and ‘Plan energy saving behavior’, *F* = 3.97, df = (3,503), *p* < 0.01. Only for the button ‘Comparison with neighbors’, univariate *F* test is insignificant, *F* = 1.76, df = (3,503), *p* = 0.15. However, for the five buttons with univariate *F* tests, *post-hoc* Bonferroni corrected pairwise tests indicate only significant differences between the predecisional stage and all other three stage groups. The means of the three stage groups, preactional, actional, and postactional, do not differ significantly. Summarizing, the analyses show that compared to the other three stage groups, people assigned to the predecisional stage rate the perceived personal relevance of all offered app buttons as the lowest. Furthermore, the analyses provide no empirical support for the expected differences in the perceived personal relevance of the offered app buttons between the other three stage groups.

Table 6 presents separately for the four stage groups the mean personal attractiveness of the app buttons perceived by the participants.

**Table 6:** Perceived attractiveness of the app buttons for the four stage groups separately.

App button	Predecision		Preaction		Action		Postaction	
	*M*	SD	*M*	SD	*M*	SD	*M*	SD
My current energy consumption	3.79	1.16	4.20	0.83	4.22	0.93	4.43	0.80
Energy saving tips	3.59	1.16	4.18	0.96	3.99	1.05	4.06	1.01
Set an energy-saving goal	3.35	1.24	3.74	0.96	3.82	1.03	3.82	1.08
My goal progress	3.28	1.22	3.81	0.93	3.81	1.00	3.78	1.11
Comparison with neighbors	2.57	1.34	2.57	1.27	2.79	1.35	2.63	1.47
Plan energy-saving behavior	3.24	1.19	3.68	1.02	3.68	1.05	3.64	1.18

Again, a MANOVA was used to compare the perceived attractiveness of the six app buttons across the four SSBC stages. The multivariate result was significant for the SSBC stage, Pillai’s Trace = 0.105, *F* = 3.02, df = (18,1500), *p* < 0.01, indicating significantly different means of the perceived relevance of the six buttons across the four SSBC stages. The univariate F tests showed that there was a significant difference between the stages for the buttons ‘My current energy consumption’, *F* = 10.28, df = (3,503), *p* < 0.01, ‘Energy saving tips’, *F* = 7.80, df = (3,503), *p* < 0.01, ‘Set an energy saving goal’, *F* = 5.50, df = (3,503), *p* < 0.01, ‘My goal progress’, *F* = 7.61, df = (3,503), *p* < 0.01, and ‘Plan energy saving behavior’, *F* = 4.62, df = (3,503), *p* < 0.01. Again for the button ‘Comparison with neighbors’, the univariate F test is insignificant, *F* = 0.75, df = (3,503), *p* = 0.52. Furthermore, the post-hoc Bonferroni corrected pairwise tests of the stage group means again indicate only significant differences between the predecisional stage and all other three stage groups. The means of the three stage groups, preactional, actional, and postactional, however, do not differ significantly again.

## 7 Discussion

This study aimed to systematically research the following three questions: (1) Could the stage-specific SSBC constructs be translated into the TaTT-MoA labels so that the TaTT can be used to identify evidence-based MoA-BCT connections? (2) How can the generally defined BCTs be translated into concrete intervention elements? (3) How can we test whether these specific intervention elements work as theoretically expected? Concerning the first question, we found that the SSBC constructs could be quite easily translated into the TaTT-MoA labels. Perhaps because the TaTT-MoA labels and definitions were extracted from 83 behavioral theories, their definitions also fit the more specific SSBC constructs. From our perspective, it would also be a real advance in environmental psychology research if we could agree on the use of consistent construct names and definitions. Often, the same names are used for differently defined constructs, or different names are used for similarly defined constructs. This inconsistency represents a barrier that should not be underestimated for the cumulative development of evaluation research in particular. In our view, however, the MoAs contained in the TaTT need to be supplemented. For example, MoAs that can be derived from social identity theory, such as social identity, group-based emotions, or collective effectiveness (Fritsche et al., 2018), are missing. Furthermore, with anticipated regret, only one emotion-related construct is included in the TaTT. In environmental psychology, many more emotions are discussed as potential MoAs, such as guilt, shame, anger, pride, joy, or being moved (e.g., Taufik and Venhoeven, 2018). A second major advantage of the TaTT is the systematic, evidence-based connection of MoAs with BCTs. Here, too, it was relatively easy for us to identify the evidence-based BCTs in the TaTT that should lead to a change in the respective SSBC constructs. In our view, environmental psychology has not yet recognized the great importance of identifying such evidence-based MoA-BCT relationships for the systematic development of evaluation research. On the one hand, there is no widely accepted classification of BCTs in environmental psychology. On the other hand, the work examining the effectiveness of various intervention techniques (e.g., Varotto and Spagnolli, 2017) focuses on their direct behavioral effect. This focus on direct behavioral effects has the disadvantage that the question of which MoA a specific intervention technique works through remains open. In our view, however, evidence-based knowledge about behavior-MoA-BCT links is important to better understand the processes on which behavior change is based. To remedy this deficit in environmental psychology intervention research, a project similar to the HBCP or SOBC project in health psychology would be very helpful.

It is interesting that the second question of how to operationalize the generally defined BCTs into concrete intervention elements is not explicitly discussed even within the HBCP literature. This is unfortunate because the question of how to operationalize abstractly defined BCTs in the context of a concrete intervention project is central. Here too, systematic compilations of already existing BCT operationalizations, as well as their critical, theoretical, and practical evaluations, are missing. Furthermore, there is little information in the literature about the creative process through which new BCT operationalizations are developed. We have not found any explanations of how to proceed methodically or how to empirically evaluate the new operationalizations before they are used in the main studies. For this reason, we explicitly described the creative development process of new BCT operationalizations in our study.

Consequently, in answering the third question, “How to test whether these specific intervention elements work as theoretically expected?,” we demonstrated how a newly developed BCT operationalization could be empirically validated in a laboratory experiment before its use in the main intervention study. The new intervention design idea consisted of a video in which a virtual agent presented and explained six app buttons summarizing different intervention packages. The conducted online study focused on empirically testing the assumption that a person’s stage membership determines their preference for the six app buttons in a theoretically expected way. However, the empirical results provided only limited support for this theoretical hypothesis: only the participants diagnosed as belonging to the predecisional stage showed the theoretically expected preference for the two app buttons ‘My energy consumption’ and ‘Energy saving tips’. Moreover, as expected, participants in this stage generally rated the personal relevance and attractiveness of all six app buttons significantly lower than participants assigned to the other three stages. This result raises doubt about whether the newly developed intervention design idea works sufficiently well. There is a high probability that the implementation of the described app-based intervention in a real, expensive intervention study would result in the finding ‘no clear intervention effect’. One possible explanation for this failure might be that dialogs used for presenting the kind of information behind the app buttons were not able to convince especially participants in the last two stages that this information fits their needs and has personal relevance for them. So, we have to return to the design table and have to develop a new, hopefully better intervention idea. Summarizing, our study results underline that the development of a theory-based intervention is obviously a complex and iterative design–evaluation–redesign–evaluation circle.

### 7.1 Limitations

We want to mention that the reliability of the discussed results of our experimental design idea test depends on the validity of our stage diagnosis. It consists of five statements from which the participant must choose one. That means that, in fact, the diagnosis is based on the selection of one item. The validity of this approach has not yet been adequately tested. Furthermore, one can criticize the artificiality of the procedure of presenting the app and Floka via a video.

### 7.2 Future research

The framework concept presented for systematic theory-driven intervention development can and should stimulate a wide range of further studies. For example, the reviewers criticize that our study does not test the Behavior-MoA-BCT chain itself. To do this, we would have to carry out a study that, in addition to evaluating the intervention design, also contains appropriate items to measure the MoAs (SSBC constructs) before and after the intervention and, if possible, a behavioral measure.

Future studies should also consider the following alternative explanations suggested by one reviewer for the non-hypothesis-consistent experimental results: Especially tenants consistently expressed their powerlessness and lack of control over actions and decisions relevant to heating and energy behaviors. This helplessness may be a reason why, in the experiment, participants were not motivated to process the provided information. Furthermore, the inconsistent experimental results could reflect participants’ interest in learning all the features of the new Floka app. From this perspective, the inconsistent results could reflect the effect of novelty or curiosity.

## Data Availability

The raw data supporting the conclusions of this article will be made available by the authors, without undue reservation.

## Footnotes

^1^ https://theoryandtechniquetool.humanbehaviourchange.org/tool
