# Correction: Combining mechanisms of action with behavior change techniques—theory-based development of an app promoting heating energy-saving behaviors

**DOI:** 10.3389/fpsyg.2025.1656691

**Published:** 2025-08-05

**Authors:** Mara Brandt, Sebastian Bamberg

**Affiliations:** ^1^Medical Assistance Systems Group, Medical School OWL, Bielefeld University, Bielefeld, Germany; ^2^Interactive Robotics in Medicine and Care, Medical School OWL, Bielefeld University, Bielefeld, Germany; ^3^Department of Social Sciences, University of Applied Sciences and Arts, Bielefeld, Germany

**Keywords:** stage model of self-regulated behavioral change, virtual agent, theory-based intervention development, mechanisms of action, behavior change techniques, theory and technique tool, menu-based, energy-saving behavior

Affiliation 2 Interactive Robotics in Medicine and Care, Medical School OWL, Bielefeld University, Bielefeld, Germany was omitted for author Mara Brandt. This affiliation has now been added for author Mara Brandt.

Author Sebastian Bamberg was erroneously assigned to affiliation 2 Interactive Robotics in Medicine and Care, Medical School OWL, Bielefeld University, Bielefeld, Germany. This affiliation has now been removed for author Sebastian Bamberg.

The original version of this article has been updated.

