# Supplementary material for: Combining mechanisms of action with behavior change techniques—theory-based development of an app promoting heating energy-saving behaviors
Source: Front Psychol. 2025 Jun 18;16:1534014. doi: 10.3389/fpsyg.2025.1534014 (PMC12213494; doi:10.3389/fpsyg.2025.1534014)
Supplement: Supplementary file 1 [file Table_1.docx]

Supplementary Material

Combining Mechanisms of Action with Behavior Change Techniques – Theory-based development of an app promoting heating energy saving behaviors

Mara Brandt*, Sebastian Bamberg

*** Correspondence:** Mara Brandt: mara.brandt@uni-bielefeld.de

# Dialog texts of the supplementary video “Virtual agent Floka explains features of an app to promote heating energy saving behavior” (https://youtu.be/Oe2VH8d2RMY?si=p6mT_C0KEFJu7m6k)

## German

Hallo, ich bin Floka. Ich freue mich darauf, dich beim Heizenergiesparen zu unterstützen.

Dafür biete ich dir verschiedene Funktionen an, die ich dir jetzt kurz erklären und zeigen möchte.

Bitte wähle dann die Funktion aus, die dich persönlich momentan am meisten interessiert.

Möchtest du herausfinden, wie viel Heizenergie du momentan verbrauchst?

Dann tippe auf "Mein Energieverbrauch".

Du siehst dann, wie viel Energie du in der letzten Woche verbraucht hast bzw. wie viel Geld oder CO2-Emissionen du einsparen kannst.

Du möchtest Heizenergie sparen, hast aber noch keine konkrete Idee wie?

Dann guck dir die Funktion "Energiespartipps" an.

Hier findest du einfache, praktische Tipps wie du mit einer Änderung deines Heiz- und Lüftungsverhaltens Heizenergie sparen kannst.

Du weißt schon, dass du Heizenergie sparen willst?

Mit der Funktion "Energiespar-Ziel setzen" kannst du für dich ein genaues Ziel setzen, wie viel Energie du im nächsten Monat oder in der gesamten Heizperiode sparen möchtest.

Du hast dir bereits Energiespar-Ziele gesetzt und möchtest deinen aktuellen Fortschritt überprüfen?

Dann wähle die Funktion "Mein Ziel-Fortschritt".

Dort erkennst du an den farbigen Kreisen sofort, wenn Handlungsbedarf besteht.

Du möchtest wissen, wie gut dein Heizverhalten im Vergleich zu deinen Nachbarn ist?

Dann wähle die Funktion "Vergleich mit Nachbarn" aus.

Je mehr goldene Sterne, desto besser bist du im Vergleich mit deinen Nachbarn.

Du siehst auch, wie viel Heizenergie du bzw. deine Nachbarn in der letzten Woche verbraucht haben.

Du hast die Absicht, Heizenergie zu sparen, aber mit der Umsetzung klappt es nicht so wie gehofft?

Unter der Funktion „Energiesparverhalten planen“ kannst du mit mir gemeinsam untersuchen, welche Hindernisse dich bisher davon abgehalten haben, deine Energiesparziele umzusetzen und einen konkreten Plan aufstellen, wie du diese überwinden kannst.

Vergisst du zum Beispiel jeden Morgen die Heizungen herunter zu drehen, bevor du zur Arbeit gehst?

Dann nimm dir doch vor, immer direkt nach dem Frühstück durch alle Zimmer zu gehen und sie herunterzudrehen.

Ich hoffe, du hast jetzt einen guten ersten Überblick über die Funktionen, mit denen ich dir helfen kann, Heizenergie zu sparen.

Wähle einfach die Funktion aus, die für dich momentan am hilfreichsten ist.

## English

Hello, I'm Floka. I look forward to supporting you in saving heating energy.

I offer you various functions for this, which I would now like to briefly explain and show you.

Please select the function that interests you the most at the moment.

Would you like to find out how much heating energy you are currently using?

Then tap on "My energy consumption".

You can then see how much energy you have used in the last week or how much money or CO2 emissions you can save.

Would you like to save heating energy, but still have no concrete idea how?

Then take a look at the "Energy saving tips" function.

Here you will find simple, practical tips on how you can save heating energy by changing your heating and ventilation behaviour.

You already know that you want to save heating energy?

With the "Set energy savings goal" function, you can set a precise goal for yourself as to how much energy you would like to save over the next month or over the entire heating period.

You have already set yourself energy saving goals and would like to check your current progress?

Then select the "My goal progress" function.

There you can see immediately from the colored circles when there is a need for action.

Would you like to know how good your heating behavior is compared to your neighbors?

Then select the "Comparison with neighbors" function.

The more gold stars, the better you are compared to your neighbors.

You can also see how much heating energy you or your neighbors have used in the last week.

Do you intend to save heating energy, but the implementation isn't going as well as you had hoped?

Under the function "Plan energy-saving behavior" you can examine together with me which obstacles have so far prevented you from implementing your energy-saving goals and draw up a concrete plan on how you can overcome them.

For example, do you forget to turn down the heating every morning before you go to work?

Then plan to go through all the rooms right after breakfast and turn them down.

I hope you now have a good first overview of the functions that I can provide to help you save heating energy.

Simply select the function that is most helpful to you at the moment.
